# Problem mobile gaming: The role of mobile gaming habits, context, and platform

**DOI:** 10.1177/14550725221083189

**Published:** 2022-04-07

**Authors:** André Syvertsen, Angelica B. Ortiz de Gortari, Daniel L. King, Ståle Pallesen

**Affiliations:** 1658 University of Bergen, Norway; 1658 University of Bergen, Norway; and University of Liège, Belgium; 1065 Flinders University, Australia; 1658 University of Bergen, Norway; and North-West University, Vanderbijlpark, South Africa

**Keywords:** gaming disorder, mental health, microtransaction, problem gaming, smartphone

## Abstract

**Aims:** Mobile gaming is a dominant form of gaming, known for its portability and for game characteristics that motivate continuous play and spending. Such involvement may also turn problematic, but research on problem gaming (PG) has tended to focus on non-mobile forms of gaming. The study was based on a cross-sectional observational design where students in upper secondary schools were recruited to a survey about mobile gaming. The age of the respondents ranged from 16 to 23 years (*n* = 519; 52.4% men; mean age = 17.2 years, *SD* = 1.1). **Methods:** We examined (1) gaming frequency, gaming contexts, and in-game spending in relation to PG; (2) gaming context in relation to academic achievement and sleep quality; and (3) PG according to gaming platform (i.e., playing on mobile, console/computer, or mixed platforms) with Kruskal−Wallis tests, chi-square tests and Spearman rank-order correlations. **Results:** PG was positively associated with mobile gaming hours per week (η^2^ = .02, *p* < .01), minutes per session (η^2^ = .03, *p* < .001), making in-app purchases (Cramer's V = .15, *p* < .05), and gaming during homework (Cramer's V = .14, *p* < .05). Statistically significant associations were found between mobile gaming in bed and later sleep midpoint for weekdays (*r_s_* = .18, *p* < .001) and weekends (*r_s_* = .11, *p* < .05). Mixed platform gamers had increased likelihood of PG, console/computer gamers had increased likelihood of being at risk for PG, and mobile gamers had lower risk for PG (Cramer's V = .18, *p* < .001). **Conclusion:** Future studies should include specific measures of mobile gaming as it appears implicated in problem gaming, albeit to a lesser degree than console and computer gaming.

Gaming on smartphones and tablets, collectively denoted as mobile gaming, has increased significantly during the last decade and is now a predominant form of gaming. Mobile gaming generated US $77.2 billion in revenue and represented 49% of the global games market in 2020 (Newzoo, 2020; Yi et al., 2019). Despite this popularity, there has been relatively little research on mobile gaming compared to non-mobile forms of gaming, including research examining adverse consequences such as problem gaming and gaming disorder (Chen & Leung, 2016; Jang & Ryu, 2016). Gaming disorder is recognised by the World Health Organization and the criteria are included in the 11th revision of the International Classification of Diseases (ICD-11), and have received strong expert agreement (Castro-Calvo et al., 2021). Although some concepts related to problem gaming, such as tolerance and withdrawal, have been the subject of debate (Paulus et al., 2018), here we use the term problem gaming to refer to a range of problematic gaming behaviours, including hazardous gaming as described in the ICD-11 (World Health Organization, 2018).

Mobile games have several structural features that tend to distinguish them from games on computers and consoles, including portability, greater emphasis on short events and easy-to-learn repeatable gameplay, and in-app purchasing options (Bowman et al., 2015). The differences between gaming platforms may limit the translational value of research on problem console and computer gaming to problem mobile gaming, where there may be, for example, more focus on avatars, narrative, complex mechanics or controls, and teamwork, and other social features (King et al., 2010). Hence, a question of interest is whether greater involvement in mobile gaming demonstrates a similar positive association to problem gaming (PG) to that observed in studies of console and computer gamers.

## Mobile gaming characteristics

Mobile games typically exhibit two fundamental characteristics − they are “casual” and “freemium” (Bowman et al., 2015). Casual games allow short play sessions, encourage replay, lack finality (i.e., an “end” of the game), and are easy to learn (i.e., low difficulty). Freemium implies free to download and play, in contrast to premium games, which cost money to download and play. Still, freemium games provide opportunities to spend money during the game to extend the experience in different ways (e.g., level boosts, game retries, fast-forwarding game processes, permanent game content). These in-game purchases enable the developers to generate revenue from an otherwise “free” experience (Alha et al., 2016; Hamari, 2015; Hamari et al., 2017). Mobile games often include structural characteristics intended to urge gamers to engage in in-app purchases. These characteristics have been termed “artificial obstacles” as they are designed to slow down game progression through time investment alone. Such features have prompted industry professionals and academics to describe mobile games as potentially predatory (King & Delfabbro, 2018, 2019) or manipulative (Alha et al., 2014; Kimppa et al., 2016; King, Delfabbro, Gainsbury, et al., 2019). Artificial obstacles may include restricting resources in the base version of the game, such as game lives or attempts, and forced waiting periods. Access to resources and rewards may be plentiful during the early engagement with a mobile game, but as time progresses, artificial obstacles become more prevalent to encourage spending. Mobile gamers who make in-game purchases often report removing obstacles or restrictions as among their primary motivations for spending money in the game (Hamari et al., 2017). However, in-game purchasing is relatively rare, with an industry report by Swrve (2019) reporting that, in 2019, only a minority (1.6%) of mobile gamers spent money on in-app purchases.

As mobile platforms allow for portable gaming, this activity might be undertaken in various contexts (e.g., when commuting, being outside of the home, or with others at social gatherings). Mobile phone gaming may distract from or otherwise interfere with normal activities, including driving (Lipovac et al., 2017), sleep (Demirci et al., 2015; Thomée et al., 2011), and academic pursuits (Kates et al., 2018). Several mechanisms have been proposed for problematic mobile phone use and related negative consequences (Billieux et al., 2015; Busch & McCarthy, 2021). Impulsivity might be especially relevant for explaining when mobile gaming leads to negative consequences (Billieux et al., 2015). The urge to alleviate boredom and receive instant gratification can drive use at inopportune times, such as during schoolwork (Busch & McCarthy, 2021). In terms of sleep, mobile gaming can displace sleep and thus lead to less sleep overall, induce arousal which prolongs sleep onset latency and impair sleep quality, and delay the circadian rhythm through evening/nocturnal bright light exposure (Cain & Gradisar, 2010). The negative consequences of mobile gaming highlight the importance of examining mobile gaming across different contexts. Studies have not yet investigated context-specific outcomes for mobile gaming, including how mobile gaming during class may relate to academic performance and/or how mobile gaming in bed may relate to sleep outcomes.

## Problem mobile gaming versus problem console and computer gaming

The relative risk of PG may depend, to some degree, on the structural characteristics of specific games, including the influence of the game platform that hosts these features. Console and computer games may contribute to PG by having games that are more varied, and complex game mechanics which require players to invest increasing amounts of time to progress and obtain desired rewards (King, Delfabbro, Perales, et al., 2019). In contrast, mobile games may contribute to PG by offering continual opportunities to play, through portability and having notifications and alerts to play (Griffiths & Nuyens, 2017). As highlighted earlier, mobile games may also employ tactics such as “artificial obstacles” that frustrate players’ desire to obtain rewards or make consistent progress, thereby requiring spending in conjunction with playing or engaging in frequent short game sessions to maximise reward payout or progress (King & Delfabbro, 2018). Research thus far suggests that both mobile and non-mobile platforms have structural characteristics associated with PG, but it is unclear whether either platform is associated with a higher risk of PG compared to the other. This section reviews the literature on video game structural characteristics that may facilitate excessive gaming that can lead to problematic gaming.

### Complex game mechanics in console and computer gaming

Some popular game genres within console and computer gaming have been associated with increased risk for PG. This includes Massively Multiplayer Online Role-Playing Games (MMORPG), First-Person Shooter (FPS) games, and Real-Time Strategy (RTS) games (Eichenbaum et al., 2015; Elliott et al., 2012; Lemmens & Hendriks, 2016; Na et al., 2017). According to King, Delfabbro, Perales, et al.’s (2019) review of player−game interactions, PG “may develop more readily and at more severe levels in relation to complex, endless, socially-driven games, irrespective of person-level characteristics” (p. 1). While mobile games may enable social gameplay, they are often relatively less sophisticated (e.g., sending invites or rewards to a friends list, as compared to continuous text-based or voice-chat-based social interaction) and designed to complement the “casual” (i.e., low effort) gameplay style (Bowman et al., 2015). It has also been proposed that mobile gaming may be less likely to elicit excessive play due to lack of features that create a sense of immersion, such as a large screen and detailed graphics and sound options (Hou et al., 2012; Jeong et al., 2016).

### Artificial obstacles and in-app purchases in mobile games

Artificial obstacles and in-app purchases are available in many consoles and computer games but appear to be more integral to the mobile game market due to its reliance on the freemium financial model (Alha et al., 2016; Alha et al., 2014; Hamari, 2015; Mäyrä & Alha, 2020). Loot boxes, a specific type of in-app purchase that features randomised rewards, have been found to be significantly more prevalent in mobile games than computer games (Zendle et al., 2020). These in-app purchases are conceptually related to artificial obstacles in that some loot boxes can include items or resources that allow the gamer to overcome them. For example, loot boxes in the mobile game *Raid: Shadow Legends* contain “champions” with randomised qualities, such that better champions allow faster game progression. Artificial obstacles can be overcome by spending money or time, and those with PG are likely to engage in both strategies more than other gamers. Spending money on in-app purchases within freemium games has been associated with PG (Dreier et al., 2017). Those with PG might be susceptible to spending money on in-app purchases/loot boxes because of increased impulsivity and need for instant gratification (Billieux et al., 2015; Busch & McCarthy, 2021). Additionally, lower punishment sensitivity and higher reward sensitivity might make those with mobile PG more willing to invest increasing time to surpass artificial obstacles and receive rewards (Raiha et al., 2020). Previous investigations suggest that structural game characteristics that facilitate prolonged use are positively associated with PG (Griffiths & Nuyens, 2017).

### Problem mobile gaming and gambling-like game mechanics

Despite their “casual” reputation, mobile games have nevertheless been implicated in excessive use and problematic play. The popular mobile game *Candy Crush*, for example, has a highly active player base. The game’s sustained popularity has been attributed to its features that resemble electronic slot machine gambling, such as frequent reinforcement and near misses, eye-catching animations, and short in-game events (Larche et al., 2017). A study by Chen and Leung (2016) estimated that 7.3% of *Candy Crush* gamers engaged in problematic play (*n* = 409). Problematic play was examined in relation to various player characteristics, including low self-control, loneliness, and boredom.

Beyond being incentives for overcoming artificial obstacles (as discussed above), loot boxes also contain gambling-like mechanics that drive engagement. In line with this, loot-box purchases are related to both PG and gambling disorder (González-Cabrera et al., 2022; Li et al., 2019). Like slot machine gambling, unpredictable reward payout can motivate loot-box purchase behaviour and chasing behaviour to win rare high-value rewards (Li et al., 2019).

Mobile PG has been associated with social anxiety, depression, loneliness, and lack of self-control (Jang & Ryu, 2016; Wang et al., 2019). It is reasonable to believe that there is an interplay between such person-level characteristics and mobile-game-level characteristics. Anxiety, depression, loneliness, and lack of self-control have all been identified as important antecedents to problematic smartphone use in a recent comprehensive review, which conceivably could extend to mobile gaming as well (Busch & McCarthy, 2021). Mobile gaming may take on a mood-regulatory function to deal with emotional difficulties and lack of self-control may predispose to more habitual use. Finally, portability could partly explain the risk of PG for mobile gaming, although there is a lack of studies examining portability and PG for mobile gaming specifically.

## The present study

Previous research has identified problematic aspects of both mobile and console/computer gaming. However, PG in the context of mobile gaming is relatively understudied. This study aims to examine (1) problem mobile gaming in relation to different types of gaming involvement, (2) mobile gaming context in relation to academic achievement and sleep quality, and (3) the association between PG and gaming platform use (mainly mobile, console/computer, or mixed platform). Gaming involvement includes gaming frequency (total hours and game session length), money spent on mobile games, in-app purchases, and mobile gaming in social, academic, and sleep-related contexts. This study also considers context-specific consequences, including mobile gaming in bed and its association with sleep onset latency, and gaming in academic situations and its association with grade point average (an indicator of academic achievement). The investigation of these variables will provide insight into different types of problem gamers according to levels of concurrent gaming engagement across platforms. We recruited adolescent/young adult upper secondary school students as problem mobile gaming appears especially relevant for this age group. Young people have the most intense gaming behaviour, show higher rates of PG, and higher rates of problem smartphone use (Csibi et al., 2021; King, Delfabbro, Perales, et al., 2019). Moreover, important potential negative effects of mobile gaming, such as mobile gaming impacting academic achievement and sleep, are highly relevant for students (Demirci et al., 2015; Kates et al., 2018; Thomée et al., 2011). Finally, various measures of mobile gaming and other gaming platforms may aid the identification of relevant associations with PG, which can be expanded upon in future research. Treatment and prevention efforts rely on accurate descriptions of relationships between players and games/game platforms (King, Delfabbro, Perales, et al., 2019). The following research questions were proposed:

1. To what extent are mobile gaming habits (gaming frequency, monetary expenditure, and gaming contexts) related to PG?
2. Is greater mobile gaming in academic situations negatively associated with academic achievement?
3. Is mobile gaming in bed prior to sleep negatively associated with sleep quality factors?
4. What type of gaming platform use (mobile, console/computer, or mixed) is associated with the highest likelihood of PG?

## Methods

### Participants and procedure

Study participants were recruited from five upper secondary schools in a major Norwegian city and invited to take part in an online survey hosted by *SurveyXact* (www.surveyxact.no). The study invitation was sent to potential participants via the students’ digital learning platforms or email addresses with the assistance of school administration staff. Potential participants were provided with a text description that informed about the study, compensation, and a link to the survey, and the associated informed consent sheet with more detailed information. The text description encouraged individuals with any form of experience with video games to participate. Data collection was conducted in April and September/October 2017. Participants were invited to participate in a raffle of 50 universal gift cards, each valued at 500 NOK (approximately €48), upon survey completion.

Inclusion criteria were being aged 16 years or older, Norwegian speaking, and having played video games at least once during the last six months. A total of 717 people provided consent and initiated the study of whom 83 had to be excluded because they were underage, did not enter age/entered unrealistic numbers, did not complete essential aspects of the survey, or provided invalid responses. Of the resulting 634 participants, 155 gave partial responses to the questionnaire. Only participants who had responded to a PG measure were included in analyses. The final sample (*n* = 519) comprised 52.4% men and age ranged from 16 to 23 years (*M* = 17.15, *SD* = 1.14).

### Measures

#### Demographic/background measures

Free-response boxes were provided for age, sex, and grade point average (GPA) for the previous semester.

#### Measures on gaming behaviour

Mobile (i.e., tablet and/or smartphone) and console/computer platforms gaming behaviours were assessed separately. Gaming frequency was assessed on ordinal five-point scales in which 1 = “daily or almost daily”, 2 = “2–4 days per week”, 3 = “about 1 day per week”, 4 = “2–3 days per month”, and 5 = “1 day per month or less” comprised the response alternatives. The main gaming platform variable was computed based on the difference in responses to this item. Equal value on both (mobile and console/computer) was coded as mixed platform use. Additionally, free-response boxes were provided for mobile weekly hours played and mobile average game session in minutes for mobile gaming. Outliers in weekly hours and average session minutes for mobile gaming were handled by winsorising, in which values above the 95th percentile were set to the 95th percentile value (for session minutes: 90; for weekly hours: 12).

#### Mobile gaming measures

Gaming in specific contexts was assessed through eight items on a five-point scale ranging from 1 = “never” to 5 = “very often”. The items included: “at school, during recess”, “at school, during class/study session”, “at home, during free time”, “at home, during schoolwork”, “while riding the bus or other forms of public transportation” and “in bed, before going to sleep” and “when with friends or acquaintances”. Free-response boxes were provided for money spent on mobile game purchases and for in-app purchases in mobile games during the last six months. Due to low variance in reported NOK, responses were dichotomised into yes/no for any money spent.

Free-response boxes were provided to report the most played mobile/tablet game during the last six months. The game was subsequently categorised as “freemium” (free to download, contains in-app purchases), “free” (free to download, no in-app purchases), or “premium” (costs money to download) based on available information on Google Play Store. Cases were treated as missing if they had invalid inputs or if the games were not available on Google Play Store.

#### The nine-item Internet Gaming Disorder Scale

The nine-item Internet Gaming Disorder Scale is a validated measure of problem gaming among adolescents and adults (Lemmens et al., 2015). The scale measures the nine criteria for the proposed diagnosis of internet gaming disorder from the Diagnostic and Statistical Manual of Mental Disorders, Fifth Edition (DSM-5) on a dichotomous yes/no rating (American Psychiatric Association, 2013). Respondents were grouped into categories based on the number of criteria endorsed: non-problem gamer (0–2 criteria), at-risk gamer (3–4 criteria), and problem gamer (5–9 criteria). The KR-20 was .76 in the present study.

#### Self-perceived gaming problem

Self-perceived gaming problem was assessed with the question: “Do you believe that you have a problem with gaming on any of the following platforms?”. Four questions were listed, each with dichotomous (yes/no) response alternatives for (1) gaming on mobile and/or tablets, (2) gaming on consoles, (3) gaming on computers, and (4) gaming on social media sites. Single-item yes/no questions for self-diagnosis of gaming problems have been shown to correlate with standardised measures of gaming problems in a clinically meaningful way (King, Delfabbro, & Griffiths, 2013).

#### Measures on sleep behaviour

Free-response boxes were provided so participants could report on time going to sleep (weekday/weekend), how long it usually takes them to fall asleep (sleep onset latency weekday/weekend), and time waking up (weekday/weekend) (Saxvig et al., 2012). Cases were treated as missing if they reported sleep onset latency exceeding their reported sleep time or had invalid responses. Outliers for sleep latency onset were handled by winsorising to the 95th percentile (for both weekday and weekend: 90 min). Sleep onset latency and the midpoint of sleep were chosen as outcome variables in analyses because longer sleep onset latency was regarded as the most proximal and plausible consequence of gaming in bed (King, Gradisar, et al., 2013). The midpoint of sleep was also chosen as it has been shown to be an indicator of chronotype, with a later midpoint indicating a delayed sleep phase (Kantermann et al., 2015). The midpoint of sleep was calculated as the midpoint between falling asleep and waking up.

### Statistical analysis

The distribution of variables was calculated for descriptives. Non-parametric techniques were used for data that were on categorical or ordinal scales, as well as continuous data due to lack of normality in the relevant instances. Chi-square tests were used to examine count differences between gaming platform groups (mainly mobile, computer/console, and mixed), gender, and PG category. When examining mobile gaming behaviour by PG category, Kruskal−Wallis tests were used for mean differences and chi-square tests for categorical outcomes. Mobile gaming context was handled categorically in these analyses, with responses “sometimes” or higher being coded true. Spearman rank-order correlation coefficients were calculated between mobile gaming in academic situations on GPA, and mobile gaming in sleep situation on sleep onset latency and the midpoint of sleep for weekdays and weekends separately.

### Ethics

Participants provided written consent in accordance with the Declaration of Helsinki (World Medical Association, 2013). No personally identifiable information was recorded. Email addresses were collected so that we were able to contact and compensate participants. Contact information was collected and stored separately from the study data.

## Results

Descriptives of the sample are presented in Table 1. There were 109 missing cases for GPA, which is likely due to first-semester students not having received their grades yet. Console/computer was the most frequent main gaming platform, followed by mobile and mixed platform use. Sex differences in gaming platform use were examined with chi-square test and revealed a statistically significant relationship *χ^2^*(*df* = 2, *n* = 519) = 87.092, *p* < .001, Cramer's V = .41. Mainly mobile gamers were predominantly female (observed: 136; expected: 87), mixed platform gamers were predominantly male (observed: 76; expected: 68), and mainly console/computer gamers were predominantly male (observed: 149; expected: 108). Among participants reporting mobile gaming, most (88%) played “freemium” games, i.e., games that are free to download but include in-app purchases.

**Table 1.** Descriptive data for the sample (*n* = 519).

		*n* (%)	M (*SD*)
Sex	Male	272 (52.4%)	
	Female	247 (47.6%)	
Main gaming platform	Mobile	183 (35.3%)	
	Mixed	130 (25.0%)	
	Console/Computer	206 (39.7%)	
Mobile game type^a^	Freemium	344 (88.0%)	
	Free	37 (9.5%)	
	Premium	10 (2.6%)	
Problem gaming	Non-problem	405 (78.0%)	
	At-risk	69 (13.3%)	
	Problem	45 (8.7%)	
Self-perceived gaming problem^b^	Mobile	19 (3.7%)	
	Console	17 (3.3%)	
	Computer	44 (8.6%)	
	Social media games	24 (4.7%)	
Age			17.15 (1.13)
Grade point average (range = 1−6)^c^			4.54 (0.72)
Sleep onset weekdays^d^			26 min (23 min)
Sleep onset weekend^d^			22 min (21 min)
Sleep midpoint weekdays^d^			3:03 (42 min)
Sleep midpoint weekend^d^			5:52 (75 min)

^a^ Valid *n* = 391, freemium is free to download and contains in-app purchases; free is fully free; premium costs money to download. ^b^Valid *n* = 512. ^c^Valid *n* = 410. ^d^Valid *n* = 411 after listwise removal, minutes are reported for sleep onset latency, hour mark reported for mean and minutes standard deviation reported for sleep midpoint.

Mobile gaming behaviour was examined by PG category. The results inform the first research question and are presented in Table 2 with associated test statistics. Kruskal−Wallis tests revealed statistically significant different ranks for hours gaming per week, *χ^2^*(*df* = 2, *n* = 396) = 12.537, *p* < .01, η^2^ = .02, likewise for minutes played during gaming session, *χ^2^*(*df* = 2, *n* = 396) = 15.319, *p* < .001, η^2^ = .03. These results indicated that mobile gaming involvement generally increases with risk severity; however, number of minutes per session was similar between those at risk and those with PG. Chi-square tests revealed statistically significant results for having spent any money on mobile in-app purchases, *χ^2^*(*df* = 2, *n* = 396) = 8.815, *p* < .05, Cramer's V = .149, and for mobile gaming during homework, *χ^2^*(*df* = 2, *n* = 396) = 7.463, *p* < .05, Cramer's V = .137. Those spending money on in-app purchases were more likely to be at risk (observed: 14; expected: 8) or addicted (observed: 8; expected: 6). Those mobile gaming while doing homework were more likely to be at risk (observed: 14; expected: 13) or addicted (observed: 15; expected: 9). The results indicate that gamers at risk or with PG were more involved with in-app purchases and mobile gaming during homework; however, differences in expected and observed frequencies were small for some comparisons.

**Table 2.** Mobile gaming behaviour according to problem gaming category (*n* = 396 after listwise removal).

	Non-problem (*n* = 303, 76.5%)	At-risk (*n* = 55, 13.9%)	Problem (*n* = 38, 9.6%)	Test statistic (*χ^2^*(df))
Hours per week (*M*, SD)	2.35 (2.97)	3.02 (3.23)	4.40 (4.28)	χ^2^(2) = 12.537**
Minutes per session (*M*, SD)	21.43 (20.73)	30.53 (26.66)	31.34 (24.68)	χ^2^(2) = 15.319**
Spent money on IAP (*n*, %)^a^	35 (11.6%)	14 (25.5%)	8 (21.0%)	χ^2^(2) = 8.815*
Spent money on game purchase (*n*, %)^a^	35 (11.6%)	13 (23.6%)	5 (13.2%)	χ^2^(2) = 5.867
Total gaming contexts (*M*, *SD*)	3.31 (2.08)	3.71 (2.18)	4.13 (2.23)	χ^2^(2) = 5.859
Gaming during recess (*n*, %)^a^	117 (38.6%)	22 (40.0%)	15 (39.5%)	χ^2^(2) = 0.044
Gaming during class (*n*, %)^a^	49 (16.2%)	13 (23.6%)	8 (21.0%)	χ^2^(2) = 2.112
Gaming at home (*n*, %)^a^	183 (60.4%)	34 (61.2%)	30 (78.9%)	χ^2^(2) = 4.960
Gaming during homework (*n*, %)^a^	61 (20.1%)	14 (25.5%)	15 (39.5%)	χ^2^(2) = 7.463*
Gaming during public transportation (*n*, %)^a^	192 (63.4%)	37 (67.3%)	28 (73.7%)	χ^2^(2) = 1.736
Gaming in the bathroom (*n*, %)^a^	177 (58.4%)	36 (65.5%)	23 (60.5%)	χ^2^(2) = 0.973
Gaming in bed before going to sleep (*n*, %)^a^	137 (45.2%)	28 (50.9%)	23 (60.5%)	χ^2^(2) = 3.477
Gaming while with friends (*n*, %)^a^	86 (28.4%)	20 (36.4%)	15 (39.5%)	χ^2^(2) = 2.973

*Note.* IPA = in-app purchase.

^a^ Percentage within each group.

*p < .05. **p < .01.

General descriptives for GPA and sleep outcomes are presented in Table 1. Spearman rank-order correlations were calculated to inform the second and third research questions (i.e., the relationship between mobile gaming in bed and sleep quality, and mobile gaming in academic situations and academic achievement). The results revealed no statistically significant relationships between mobile gaming during class and GPA (*r_s_* = −.035, *n* = 323, *p* = .53) or mobile gaming during homework and GPA (*r_s_* = .053, *n* = 323, *p* = .37). There were no statistically significant relationships between mobile gaming in bed and sleep onset latency on weekdays (*r_s_* = .071, *n* = 389, *p* = .16) or weekend (*r_s_* = .049, *n* = 385). There were, however, statistically significant relationships between mobile gaming in bed and the midpoint of sleep for weekdays (*r_s_* = .182, *p* < .001, *n* = 373) and weekends (*r_s_* = .114, *p* < .05, *n* = 357), suggesting mobile gaming in bed was associated with delayed sleep phase on both weekdays and weekends.

Finally, PG, self-perceived gaming problem, and the main gaming platform was investigated. Gender differences in PG were examined with chi-square test, revealing a statistically significant relationship *χ^2^*(*df* = 2, *n* = 519) = 20.499, *p* < .001, Cramer's V = .199. Those in the non-problem category were more predominantly female (observed: 214; expected: 193). Males were more likely to be in the at-risk category (observed: 50; expected: 36) and the PG category (observed: 31; expected: 24). Playing on a computer was the most frequent platform associated with self-perceived gaming problem, followed by social media games, mobile games, and console games (see Table 1). A chi-square test was conducted on main (i.e., most played) gaming platform by PG category, which indicated a statistically significant relationship, *χ^2^*(*df* = 4, *n* = 519) = 33.355, *p* < .001, Cramer's V = .179. Those who played mainly mobile games were less likely to be at risk (observed: 12; expected: 24) or addicted (observed: 7; expected: 16). Main console/computer gamers were more likely to be at risk (observed: 41; expected: 27). Mixed platform gamers were more likely to be addicted (observed: 22; expected: 11). Regarding the fourth research question, the results indicate that mixed platform gamers have the highest likelihood of PG and that console/computer gamers are at increased risk. Mainly mobile gaming carried a reduced likelihood of being at risk or having PG.

## Discussion

The aim of the present study was to examine problem mobile gaming, with a focus on gamers’ habits, contexts of gaming, and the consequences of gaming in these contexts. The study was also aimed to investigate PG according to gaming platform preference (mainly playing on mobile, console/computer, or mixed platforms). Participants were classified as having no gaming problems (78.0%), being at risk of PG (13.3%), or reporting PG (8.7%). These figures were higher than the reported global prevalence rates of PG of 1.96% overall and 4.6% among adolescents (Fam, 2018; Stevens et al., 2021). The higher rate of PG in the present study should not be considered a prevalence rate and was likely influenced by the younger age of the sample and purposive sampling of gamers.

The study results indicated that both mobile gaming hours per week and minutes per mobile game session were positively associated with PG. This association had a small effect size and was consistent with previous studies showing that gaming frequency positively correlates with PG (King, Delfabbro, et al., 2019). Most participants who engaged in mobile gaming reported that they mainly played a “freemium” mobile game, which suggests that they were exposed to (if not directly interacting with) game characteristics common to these types of mobile games, including artificial obstacles and in-app purchase options. Interestingly, spending money on mobile in-app purchases was more likely to be reported by those who reported PG in the present study. Previous studies also link monetary expenditure in games to both PG and gambling disorder (Brooks & Clark, 2019; Dreier et al., 2017; Li et al., 2019).

Mobile gaming during class and while doing homework were not found to be significantly related to academic achievement. This finding differs from the results of a recent meta-analysis where forms of mobile phone engagement such as binary exposure/non-exposure, problem use, frequency, and total time spent were inversely associated with academic achievement such as test scores, GPA, and learning task performance (Kates et al., 2018). However, the meta-analysis also found smaller effect sizes for studies using cross-sectional rather than experimental designs, studies using frequency scales rather than binary constructs, and students at K-12 education level compared to university level. These characteristics match the present study, which, combined with a reduced number of participants for the relevant analyses, may account for the lack of an association.

We examined associations between mobile gaming in bed and sleep onset latency and midpoint for sleep. Unexpectedly, no association was found between mobile gaming in bed and sleep onset latency, which was assumed to be the most direct way mobile gaming could influence sleep (i.e., displacement: the person stays up late to play mobile games). However, mobile gaming in bed was weakly associated with later midpoint for sleep, which reflects a delayed sleep phase on weekdays and weekends (Kantermann et al., 2015). When accompanied by obligations to get up early (e.g., school), the delayed sleep phase may affect daytime functioning as this is usually a consequence of curtailed sleep. The association between mobile gaming in bed and later midpoint for sleep was strongest for weekdays, which is notable as delayed sleep likely confers more problems on weekdays due to morning-time obligations the following day. The delayed sleep phase could be due to mobile gaming delaying the circadian rhythm through evening light exposure (Cain & Gradisar, 2010). This is problematic as adolescents already show higher rates of delayed sleep phase disorder (DSPD) compared to adults (Sivertsen et al., 2013). Gaming can also negatively influence sleep by increasing arousal, which could make those who are more motivated by rewards and/or stimulation particularly vulnerable (Cain & Gradisar, 2010). Relatedly, both PG and attention-deficit hyperactivity disorder (ADHD) are associated with DSPD (Ko et al., 2020; Sivertsen et al., 2015). Future research should investigate the potential mediating role of player motivation for stimulation and rewards on mobile gaming in bed.

The study also investigated types of gaming platforms and their association with the risk of PG. The results indicated that participants who reported mixed platform gaming were more likely to report PG; those whose main platform was a console or computer were more likely to be at risk of PG, while those who mainly played on mobiles or tablets were at reduced risk (i.e., lower PG scores). Those who play on multiple platforms may generally be more involved in gaming and prioritise gaming activities. Relatedly, playing a larger number of game genres has been found to predict PG (Donati et al., 2015). This finding also mirrors the gambling literature in which the number of gambling activities predicts problem gambling (Brosowski et al., 2012; LaPlante et al., 2014).

Playing mainly console and computer games carried an increased likelihood of being at risk of PG. Participants also reported self-perceived gaming problems according to gaming platforms. Self-perceived gaming problems/addiction have previously been examined in a nationally representative Polish study by Lewczuk et al. (2021) which found that 4.2% of participants perceived themselves as game addicts (all platforms). This is comparable to our findings on self-perceived gaming problems on mobiles (3.7%), consoles and (3.3%), and social media (4.7%), although we recruited a purposive sample of gamers. Problematic computer gaming was the most frequently reported self-perceived problem in the present study (8.6%). This finding might be explained by computer games being more varied and complex through characteristics such as player progression options, social interaction, story elements, and realistic graphics. For instance, MMORPGs are more prevalent within computer gaming and have been more commonly associated with PG (King, Delfabbro, et al., 2019); however, it bears noting that game genres such as Multiplayer Online Battle Arena (MOBA) and Battle Royale games (e.g., *Fortnite*) have become more popular over the last five years. Seeing gaming as morally wrong predicts higher levels of self-reported PG (Lewczuk et al., 2021). It is possible that moral incongruence is higher among computer gamers, leading to higher frequency of self-perceived gaming problems. Computer gamers might identify more with the “gamer” label due to the more involving nature of their gaming platform and thus be more likely to internalise public stigma against gaming (Peter et al., 2019; Stone, 2019).

Overall, mobile gaming was associated with a lower risk of PG compared to the other platforms. This may be partly due to the “casual” nature of mobile games. Another explanation may be that the profile of those who prefer mobile gaming differs from those who play computer/console games. For instance, female gamers were significantly more likely to be mobile gamers and less likely to be at risk or have PG. Conversely, being male was associated with being at risk or having PG − a well-established association in the literature (Mihara & Higuchi, 2017; Paulus et al., 2018). It is possible that mobile gaming does not carry a reduced risk for PG per se, but, rather, is more prevalent among female gamers who are also at reduced risk of PG. Furthermore, the potential for PG in mobile gaming was supported by some participants self-perceiving a mobile gaming problem and by specific mobile gaming habits being associated with PG.

### Strengths and limitations

The present study shares common limitations associated with cross-sectional studies using convenience sampling. Causality cannot be ascertained, generalisability may be limited, and participants’ reports are potentially subject to recall and social desirability biases. A larger sample size could have increased the statistical power to identify potential effects that were not detected. Table 2 suggests differences between non-problem gamers, those at risk, and those having PG for outcomes such as total mobile gaming contexts and mobile gaming before going to sleep. However, the reduced sample size was somewhat mitigated by the relatively high number of participants being at risk (13.3%) or having PG (8.7%), which strengthened statistical power in the relevant analyses. The measure of academic achievement was limited by participants reporting on past-semester GPA. The measure of current mobile gaming may not provide representative responses when participants’ mobile gaming habits are unstable. We also lacked a validation measure of GPA; desirability bias might lead participants to report more favourable grades. However, reported GPA in our sample (*M* = 4.54, *SD* = 0.72) was comparable to that collected through school registries in a large study of Norwegian upper secondary school students (*M* = 4.10, *SD* = 0.79) (Sæle et al., 2016). Finally, the measure of problem gaming may not ideally capture the criteria and guidelines reported in the ICD-11 (King et al., 2020).

The present study examined a form of gaming that is understudied, and expanded upon previous studies on mobile gaming by including varied measures of mobile gaming habits, examining the relationship between mobile gaming and PG, and comparing mobile gaming to other gaming platforms (Chen & Leung, 2016; Jang & Ryu, 2016; Lopez-Fernandez et al., 2018). Another strength of the present study was the roughly equal representation of male (52.4%) and female (47.6%) gamers. The underrepresentation of female gamers has been a common limitation in PG research (King & Potenza, 2020).

## Conclusion

Playing on multiple platforms was most strongly positively associated with PG, followed by mainly playing console/computer games. These results indicate that researchers and clinicians should pay attention to the variety of gaming platform engagement in addition to gaming frequency and specific game types/game genres. While playing mainly mobile games was found to carry a reduced risk of PG, researchers would be remiss to overlook this type of gaming in future research on PG. Mobile gaming frequency, mobile game session length, and engagement with in-app purchases were positively associated with PG. Future studies could benefit from including additional measures of mobile gaming to explore its potential function in combination with other gaming activities. Future studies may consider including detailed measures such as in-app purchases by assessing various types of buying goods and services (e.g., purely cosmetic upgrades and upgrades that make the player more likely to win).

The negative consequences of problematic mobile gaming were found to be mixed. Mobile gaming in bed was associated with delayed sleep, however, gaming during class/during homework was not related to GPA. These results highlight the need for more sophisticated approaches to the measurement of gaming and how it may interfere with daily functioning. Some effects may be relatively small and occur only under certain conditions, and thus may be difficult to detect in relatively small exploratory studies (Kates et al., 2018).

This study provides empirical data on mobile and non-mobile gaming, contributing to continuing discussion of different forms of problem gaming as described in the DSM-5 and ICD-11. Mobile gaming represents the most popular form of gaming but has received less research compared to console and computer gaming (Yi et al., 2019). Mobile gaming is likely to continue to grow and expand its audience as more people adopt smartphones from a young age. It is important for researchers to study the uniqueness of mobile gaming characteristics to help develop a more nuanced and complete understanding of gaming overall and its potential positive and negative effects.
